# Disaggregation of canopy photosynthesis among tree species in a mixed broadleaf forest

**DOI:** 10.1093/treephys/tpae064

**Published:** 2024-06-12

**Authors:** Marko Stojanović, Georg Jocher, Natalia Kowalska, Justyna Szatniewska, Ina Zavadilová, Otmar Urban, Josef Čáslavský, Petr Horáček, Manuel Acosta, Marian Pavelka, John D Marshall

**Affiliations:** Global Change Research Institute, Czech Academy of Sciences, Bělidla 4a, Brno 603 00, Czech Republic; Global Change Research Institute, Czech Academy of Sciences, Bělidla 4a, Brno 603 00, Czech Republic; Thünen-Institut für Agrarklimaschutz Bundesallee 68 38116 Braunschweig Germany; Global Change Research Institute, Czech Academy of Sciences, Bělidla 4a, Brno 603 00, Czech Republic; Global Change Research Institute, Czech Academy of Sciences, Bělidla 4a, Brno 603 00, Czech Republic; Global Change Research Institute, Czech Academy of Sciences, Bělidla 4a, Brno 603 00, Czech Republic; Global Change Research Institute, Czech Academy of Sciences, Bělidla 4a, Brno 603 00, Czech Republic; Global Change Research Institute, Czech Academy of Sciences, Bělidla 4a, Brno 603 00, Czech Republic; Global Change Research Institute, Czech Academy of Sciences, Bělidla 4a, Brno 603 00, Czech Republic; Global Change Research Institute, Czech Academy of Sciences, Bělidla 4a, Brno 603 00, Czech Republic; Global Change Research Institute, Czech Academy of Sciences, Bělidla 4a, Brno 603 00, Czech Republic; Global Change Research Institute, Czech Academy of Sciences, Bělidla 4a, Brno 603 00, Czech Republic; Department of Forest Ecology and Management, Swedish University of Agricultural Sciences, Umeå 90183, Sweden; Leibniz-Zentrum für Agrarlandschaftsforschung, Isotope Geochemistry and Gas Fluxes, Müncheberg 15374, Germany; Department of Geological Sciences, Box 460, Gothenburg University, Gothenburg 40530, Sweden

**Keywords:** deciduous angiosperms, eddy covariance, gross primary production, phenology, phloem isotopes, sap flow

## Abstract

Carbon dioxide sequestration from the atmosphere is commonly assessed using the eddy covariance method. Its net flux signal can be decomposed into gross primary production and ecosystem respiration components, but these have seldom been tested against independent methods. In addition, eddy covariance lacks the ability to partition carbon sequestration among individual trees or species within mixed forests. Therefore, we compared gross primary production from eddy covariance versus an independent method based on sap flow and water-use efficiency, as measured by the tissue heat balance method and δ^13^C of phloem contents, respectively. The latter measurements were conducted on individual trees throughout a growing season in a mixed broadleaf forest dominated by three tree species, namely English oak, narrow-leaved ash and common hornbeam (*Quercus robur* L., *Fraxinus angustifolia* Vahl, and *Carpinus betulus* L., respectively). In this context, we applied an alternative ecophysiological method aimed at verifying the accuracy of a state-of-the-art eddy covariance system while also offering a solution to the partitioning problem. We observed strong agreement in the ecosystem gross primary production estimates (R^2^ = 0.56; *P* < 0.0001), with correlation being especially high and nearly on the 1:1 line in the period before the end of July (R^2^ = 0.85; *P* < 0.0001). After this period, the estimates of gross primary production began to diverge. Possible reasons for the divergence are discussed, focusing especially on phenology and the limitation of the isotopic data. English oak showed the highest per-tree daily photosynthetic rates among tree species, but the smaller, more abundant common hornbeam contributed most to the stand-level summation, especially early in the spring. These findings provide a rigorous test of the methods and the species-level photosynthesis offers avenues for enhancing forest management aimed at carbon sequestration.

## Introduction

Forests provide an important sink for atmospheric carbon dioxide (CO_2_), a greenhouse gas that contributes to ongoing climate change (Pan et al. 2011). Particularly important is canopy photosynthesis, or gross primary production (GPP), which measures the total rate of CO_2_ uptake from the atmosphere. Ecosystem-scale CO_2_ fluxes are generally measured using the eddy covariance (EC) method (Baldocchi 2003; Aubinet et al. 2012). The key strength of this method is that it integrates CO_2_ fluxes over everything that happens below a horizontal plane above the canopy (Baldocchi 2008). Consequently, its results can accurately assess the net CO_2_ balance of the whole ecosystem.

Despite its strengths, the EC method faces challenges in describing the component fluxes occurring within the ecosystem (Wehr et al. 2016), as it typically involves using quasi-mechanistic models to distinguish, e.g. GPP from ecosystem respiration (R_eco_) (Reichstein et al. 2005). Specifically, it is often assumed that GPP can be estimated from an asymptotic relation with incoming radiation and that R_eco_ can be estimated from temperatures. If either of these models is incorrect, both the GPP and the R_eco_ estimates will be inaccurate, and our understanding of the ecosystem will be flawed. Measurements by the EC method are sometimes compared with ground-based biometric measurements, including chamber measurements of component flux and allometric measurements of component mass (Ryan 2023). Typically, the EC method reports lower R_eco_ and higher net annual C storage (i.e. net ecosystem production) compared with biometric estimates (Campioli et al. 2016; Marshall et al. 2023). Another challenge, especially in forests, is that the elevated canopy and divergence in stratification across the canopy partially block the passage of eddies from the air mass below the canopy into the air mass above, termed decoupling (Thomas et al. 2013). This limits the ability of the below-canopy CO_2_ sources to contribute to measurements made above and is especially pronounced in complex terrain (Jocher et al. 2020) and forests with diverse structure (Paul-Limoges et al. 2017; Kowalska et al. 2022).

Moreover, EC cannot disaggregate the ecosystem fluxes among individual trees or species within the ecosystem (cf. Kowalska et al. 2020). Understanding the contribution of each species to overall CO_2_ sequestration is vital for effective forest management, especially in the context of climate-change mitigation strategies (Campioli et al. 2015; Luyssaert et al. 2018). Individual trees can vary greatly in their rates of photosynthesis, and hence their carbon sequestration potential, due to differences in factors such as species, age, health status and environmental conditions (Bassow and Bazzaz 1997). Several studies have tried to elucidate the relationship between EC-derived photosynthesis (carbon source) and carbon allocation (carbon sink), typically focusing only on aboveground woody biomass (Delpierre et al. 2016; Krejza et al. 2022; Puchi et al. 2023). Although such studies can allocate wood growth among species and over the season, they cannot provide species-specific rates of photosynthesis because the proportion of photosynthesis used for wood growth is small and variable (e.g. Marshall et al. 2023). Therefore, a method that can accurately estimate the photosynthetic rates of individual trees, and thereby their CO_2_ sequestration potential, could greatly enhance our ability to manage forests for carbon capture (Ameray et al. 2021).

We tested GPP estimates by EC against an independent, alternative ecophysiological method (Hu et al. 2010; Klein et al. 2016). The ecophysiological method (ISO/SF) relies on measurements of sap flow as a proxy of transpiration (Poyatos et al. 2021). When combined with the atmospheric vapor pressure deficit (VPD), transpiration yields estimates of stomatal conductance (Jones 2013). The method also relies on stable carbon isotope ratios (δ^13^C) of organic compounds dissolved in the phloem sap, which can be used to estimate intrinsic water-use efficiency (iWUE), the ratio of CO_2_ uptake to stomatal conductance. The δ^13^C of phloem contents has proven useful for this purpose (Vernay et al. 2020; Schiestl-Aalto et al. 2021) because they provide a weighted estimate of iWUE of recent gas exchange based on all the dissolved carbon transported through the phloem (Gimeno et al. 2021). Notably, both measurements are made on tree stems, allowing for the disaggregation of the canopy flux down to individual trees. The per-tree scale gives us the opportunity to assess the contributions of different species and size classes to the canopy-scale GPP estimate derived from EC. Finally, ISO/SF method can be applied to all types of forest ecosystems, including those in complex terrain and with decoupling issues (Etzold et al. 2010; Jocher et al. 2017, 2020).

In previous studies, the ISO/SF method agreed well with EC in a boreal pine forest (Vernay et al. 2020), but significantly underestimated GPP by EC in mixed forest of pines and spruces (Vernay et al. 2024). Evidently, the Granier heat dissipation (GHD; Granier 1985) method used for sap flow measurements in these studies worked well for pine, but not for spruce (Vernay et al. 2024). The underestimates were likely due to problems with scaling measurements to the whole stem (Steppe et al. 2010; Peters et al. 2018). Similar underestimations by the GHD method have been previously reported in Aleppo pines (Klein et al. 2016). Here we have applied an alternative sap flow method, namely the Tissue Heat Balance (THB) method, which is thought to minimize scaling issues (Schulze et al. 1985; Urban et al. 2012).

In this study, we compared methods in a mixed floodplain forest dominated by three deciduous broadleaf tree species: English oak (*Quercus robur* L.), narrow-leaved ash (*Fraxinus angustifolia* Vahl) and common hornbeam (*Carpinus betulus* L.), hereafter referred to as oak, ash and hornbeam, respectively. Oak and ash have ring-porous wood, and hornbeam has diffuse-porous wood; both these are quite different from the tracheids of conifers that have previously been studied. To our knowledge, this is the first study to test GPP estimates by the two methods in a mixed broadleaf ecosystem and the first to use the THB method for sap flow. Given the focus here on broadleaf species, we expected that the impact of leaf development on photosynthesis would be crucial. Therefore, we provide detailed measurements of leaf phenology to help explain photosynthesis dynamics in these species.

In summary, the objectives of the study were: (i) to compare GPP estimates from EC techniques to independent estimates based on sap flow and phloem δ^13^C for the first time in a diverse, deciduous mixed-broadleaf forest, (ii) to apply the THB method of estimating sap flow for the first time to ISO/SF GPP estimation and (iii) to partition canopy GPP among three phenologically distinct species over the course of the growing season.

## Materials and methods

### Site description

The Lanžhot site is a part of the Integrated Carbon Observation System (www.icos-cp.eu/news-and-events/icoscapes/lanzhot) network, representing the hardwood floodplain forests (Kowalska et al. 2020). It is located in the south-east part of the Czech Republic, ~6 km north of the confluence of the Morava and Dyje rivers (48° 40.090 N, 16° 56.780 E; 154 m a.s.l.). The long-term average annual precipitation from 1961 to 2017 is around 497 mm, with a mean annual temperature of 9.7 °C. The average groundwater level during 1966 to 2018 was −2.6 m (Szatniewska et al. 2022). The plot is on an alluvial plain, and the main soil types are Eutric Humic Fluvisol, Haplic Fluvisol and Eutric Fluvisol with a minimum soil depth of 60 cm (Acosta et al. 2017).

The experimental site, consisting of ~ 110-year-old trees, is composed of typical hardwood species representative for the region (Szatniewska et al. 2022). This forest is predominantly composed of oak, ash and hornbeam, with a range of other species contributing to the biodiversity but having a negligible contribution to the stand’s basal area (see Table S1 as Supplementary data at *Tree Physiology* Online).

The average stand height is 27 m. Oak and ash create the main stand canopy and represent dominant and co-dominant social classes, while hornbeam is primarily found under the main canopy layer (see Figs S1 and S2 as Supplementary data at *Tree Physiology* Online). Despite its small size, hornbeam stems are most numerous, followed by ash and oak (see Table S1 as Supplementary data at *Tree Physiology* Online).

### Environmental data and leaf phenology observations

**Figure 1:**
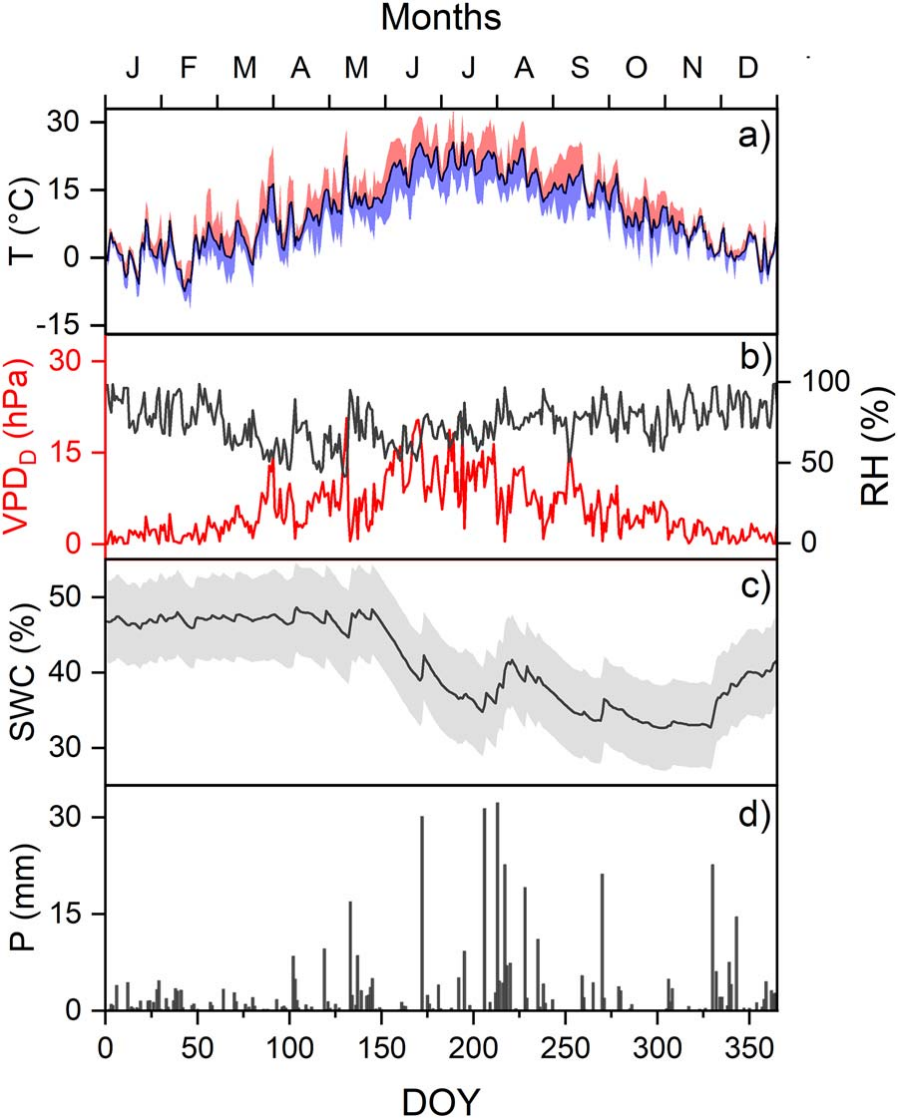
Weather at the Lanžhot site in 2021 vs. day of year (DOY). (a) Daily mean (solid line), maximum (upper band) and minimum (lower band) air temperature (T; °C), (b) VPD_D_ (hPa) and relative air humidity (RH; %), (c) daily SWC (%) with band indicating standard error of mean among sensors, and (d) daily precipitation sums (P; mm).

Although microclimatic conditions on this site have been recorded since 2015, this study focused specifically on data from 2021 (Fig. 1).Throughout this year, relative air humidity (RH, %) and air temperature (T, °C) were recorded with calibrated EMS 33 sensors (EMS Brno, Czech Republic), while precipitation (P, mm) was measured using a rain gauge 386C (Met One Instruments, Grants Pass, OR, USA). Global incoming radiation (R_g_, W m^−2^) was measured with a CNR4 (Kipp & Zonen, Delft, The Netherlands) radiation sensor. Photosynthetic Photon Flux Density ( μmol m^−2^ s^−1^) was measured under the canopy with quantum EMS 12 (EMS Brno, Czech Republic) sensors. Atmospheric pressure (P_a_, hPa) was recorded at 3 m of height with a barometer PTB110 (Vaisala, Finland).The VPD (hPa) was calculated from RH and T, by first determining the saturation vapor pressure, and then calculating the difference between the actual and the saturation RH (Jones 2013). The soil water content (SWC, %) was assessed at various soil depths—5, 10, 20, 50 and 100 cm—in each of the four cardinal directions around the plot center, using CS616l sensors (Campbell Scientific, Inc., USA). All data were recorded every 30 s, with an averaging period of 30 min.

The atmospheric CO_2_ concentration (C_a_, p.p.m.) and atmospheric δ^13^C signature (δ^13^C_a_, ‰; see Fig. S3 as Supplementary data at *Tree Physiology* Online)in 2021 were both collected from the National Oceanic and Atmospheric Administration database using the nearest sample station at the Ochsenkopf in Germany (Thompson et al. 2009).

**Figure 2:**
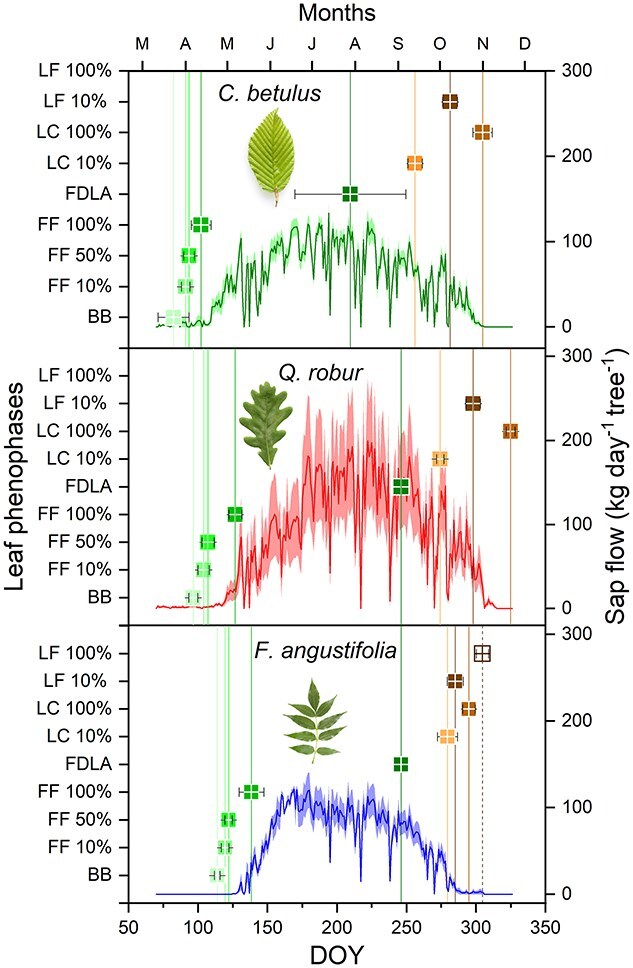
Seasonal dynamics of leaf phenological phases and sap flow in hornbeam, oak, and ash during 2021 vegetative season. BB = budbreak, FF 10–100% = foliage formation, FDLA = fully developed leaf area, LC 10–100% = leaf coloring, LF 10–100% = leaf fall. Whiskers and bands are standard deviations of leaf phenological observations (*n* = 7 per species) and sap flow (*n* = 5 per species), respectively.

Leaf phenological observations were conducted on the dominant tree species at the study site (Fig. 2). Observations were focused on seven oak trees, five ash trees and six hornbeam trees, each was chosen for proximity to the meteorological mast. Consistent observations were carried out by the same person three times per week in the spring, and twice per week during summer and autumn. Using binoculars, these observations were made from both the ground and from the mast, primarily targeting the upper parts of the tree crowns. Using the methodology previously described in Nezval et al. (2020), leaf phenophases were classified into several stages: bud break (BB), 10 to 100% foliage formation (FF), fully developed leaf area (FDLA), 10% leaf color change (LC10), 10% leaf fall (LF10), 100% leaf color change (LC100) and 100% leaf fall (LF100) with the latter observed only in ash trees.

### Eddy covariance GPP derivation

The energy and matter fluxes at Lanžhot were determined by the EC method. The EC system was installed on the meteorological mast at 44 m above the ground. It consisted of an ultrasonic anemometer (Gill Instruments, Hampshire, UK) for measuring the 3D wind components and sonic temperature, as well as an enclosed infrared gas analyzer LI-7200 (LI-COR, Lincoln, NE, USA) for CO_2_ and water vapor concentration measurements. All measurements were taken at a frequency of 20 Hz.

A second identical system was installed under the canopy, positioned 3.5 m above the ground, near the meteorological mast (Kowalska et al. 2022). The below-canopy data were used to estimate the extent of canopy decoupling by investigating the relation of the standard deviation of vertical wind (σ_w_) between below and above canopy air masses. In the earlier study, decoupling events occurred frequently but had little influence on annual net ecosystem CO_2_ exchange estimates, meaning that our study site was relatively free of such errors. We did not use the decoupling filter in our analysis because it tended to eliminate morning and evening values. These values were necessary for accurate daily sums. Daily sums were necessary to match the sap-flow data in the ISO/SF method (described below).

Vertical turbulent fluxes were computed by the EC method described previously by, e.g. Lee et al. (2005) and Aubinet et al. (2012), with an averaging period of 30 min. All flux calculations were performed with the EddyPro EC software (Fratini and Mauder 2014). The accurate application of the EC method requires several corrections to be made on the calculated covariance between the vertical wind component (*w*) and the quantity of interest, for instance, CO_2_ when considering CO_2_ flux. A planar fit coordinate rotation, as detailed by Wilczak et al. (2001), was employed to ensure the long-term average of the vertical wind measurements was negligible, as well as to rotate the measurements into the main wind direction. Moreover, corrections for potential flux losses in the high-frequency range, possibly due to path length averaging or the spatial separation of the sonic measurement path and gas analyzer, were applied following the procedure described in Ibrom et al. (2007).

The EC software also included a quality-flagging scheme, testing the data for stationarity and development of turbulence; it provided an overall quality flag combining the results of both tests (Foken et al. 2005). Only flux data with best quality were used for further considerations. We also applied a friction velocity (u_*_) threshold on our flux data, which ensured that the mixing across the canopy was sufficient to measure representatively above the canopy.

Carbon flux partitioning and data gap filling were performed using R package REddyProc (Wutzler et al. 2018). Gap-filling was conducted there via marginal distribution sampling (Reichstein et al. 2005). Regarding the flux partitioning, we followed the ‘daytime’ approach as proposed by Lasslop et al. (2010). This approach uses night-time CO_2_ flux to infer the temperature sensitivity of R_eco_ but uses daytime data only to parameterize the light- and temperature-driven models of GPP (hereafter GPP_EC_; g C m^−2^ d^−1^) and R_eco_, respectively (Wohlfahrt and Galvagno 2017).

### Tree level transpiration and conductance

Sap flow was measured in five to seven healthy, undamaged trees of each species situated within the footprint area of the EC system measurements. The sampled trees were selected to represent the distribution of diameters at breast height across the forest (see Fig. S2 as Supplementary data at *Tree Physiology* Online; Szatniewska et al. 2022). The sap flow measurements were conducted using the THB method with internal heating and sensing (Kučera et al. 1977; Čermák et al. 2004), which allowed approximation of whole-tree water use or tree transpiration. The specific sensors employed were the EMS81 modules (EMS Brno, Czech Republic), which were installed at breast height of the trees (1.3 m), typically on the north side to avoid interference with phloem sampling. Contrary to other methods, THB involves heating the xylem with an alternating electrical current passed between a set of 3 flat stainless steel electrodes. A major advantage of this approach is that it eliminates heat transfer between the heating element and the water-active xylem, significantly reducing errors at higher sap flow rates (Tatarinov et al. 2005). Moreover, when the electrode length adequately matches the expected sapwood depth, the measurements become almost independent of the radial profile of sap flow rates. Consequently, we utilized shorter electrodes for ring-porous species (oak and ash) and longer electrodes for the diffuse-porous species (hornbeam), corresponding to the deeper sapwood typically found in diffuse-porous species. Based on this assumption, the sensors provide sap flow values in kilograms per cm of stem circumference at the cambium.

Sap flow data were recorded at 2-min intervals and stored as 10-min averages. These values were then further averaged to hourly intervals and expressed as specific sap flow (Q) per unit of trunk circumference (kg h^−1^ cm^−1^). The Exponential Feedback Weighting method as detailed by Kučera et al. (2020) was used to eliminate heat losses by establishing a baseline (using 5-day period) from the night-time zero-flow values when there was no demand for evaporation. Hourly Q values were further aggregated to daily sums per tree (kg day^−1^ cm^−1^). Finally, to compute the sap flow for the entire tree (Q_tree_, kg day^−1^ tree^−1^), Q was multiplied by the specific circumference of each individual tree, excluding the bark and phloem layers, the thickness of which was measured during sensor installation (Fig. 2).

Crown conductance to H_2_O vapour was then calculated using the following equation (Vernay et al. 2020):


(1) \begin{equation*} {g}_{\mathrm{tree}}=\frac{\frac{Q_{\mathrm{tree}}}{M_{H_2O}}\times 1000}{\frac{VPD_D}{P_a}}.\end{equation*}

In Eq. (1), *Q*_tree_ is first converted from kg H_2_O d^−1^ tree^−1^ to mol H_2_O day^−1^ tree^−1^. *VPD_D_*, representing the daytime vapor pressure deficit, was calculated using the periods when *R_g_* exceeded a threshold of 10 W m^−2^ to define ‘daytime’ and divided by barometric pressure (*P_a_)* to convert to mole fraction. The resulting conductance values are expressed in mol H_2_O tree^−1^ day^−1^, meaning that they integrate over all the leaves in the crown of a given tree on a given day.

### Phloem isotopes, discrimination and ISO/SF GPP derivation

We quantified the δ^13^C values of the solutes transported within the phloem tissue, represented as δ^13^C_p_, in per mil (‰). Phloem samples were collected biweekly from five trees of each species, within the same trees used for sap flow measurements (see Fig. S2 as Supplementary data at *Tree Physiology* Online), beginning on 31 March and ending on 2 November 2021, to encompass the entire photosynthetic period. A bark punch, 9 mm in diameter, was used to extract a cylinder containing bark, phloem, cambium and xylem. To minimize local disturbance, samples were taken from slightly different heights on the trees at each sampling. After removing bark and xylem, a sample containing active phloem was subsequently dropped into a 2 mL vial containing 1 mL of deionized water, allowing time for exudation (approx. 5 h; Gessler et al. 2004). The phloem sample was then removed from the vial, and the exudates were frozen until the processing time.

To determine δ^13^C values, 150 μL of phloem exudates were transferred and dried in tin capsules at 60 °C for 12 h, following the method of Gessler et al. (2004), with minor modifications as detailed in Vernay et al. (2020). For δ^13^C measurements, the samples were combusted to CO_2_ at 960 °C using an elemental analyzer varioPYRO cube (Elementar Analysensysteme, Germany). The stable isotopes in the resulting CO_2_ were then determined by a continuous flow isotope ratio mass spectrometer ISOPRIME100 (Isoprime, UK). Finally, the δ^13^C_p_ values were calculated as the deviation from the Vienna Pee Dee Belemnite (VPDB) standard using the formula,


(2) \begin{equation*} \mathrm{\delta} ^{13}\mathrm{C}_{\mathrm{p}}=\left[\frac{\left(\frac{^{13}\mathrm{C}}{^{12}{\mathrm{C}}_{\mathrm{phloem}}}\right)}{\left(\frac{^{13}\mathrm{C}}{^{12}\mathrm{C}_{\mathrm{VPDB}}}\right)}- 1\right]\times 1000,\end{equation*}

where δ^13^C_p_ is the ratio of the heavy to light isotope (^13^C/^12^C) in phloem sample.

The ^13^C discrimination (Δ, ‰) was estimated by correcting the δ^13^C_p_ values in phloem exudates for the δ^13^C_a_ values at the time of photosynthesis. It was assumed that the phloem exudates were predominantly composed of recent photosynthetic carbohydrates (Klein et al. 2016; Vernay et al. 2020). The following equation was utilized for the calculations:


(3) \begin{equation*} \Delta =\frac{\mathrm{\delta}^{13}{\mathrm{C}}_{\mathrm{a}}-{\mathrm{\delta}}^{13}{\mathrm{C}}_{\mathrm{p}}}{1+\frac{\mathrm{\delta}^{13}{\mathrm{C}}_{\mathrm{p}}}{1000}}. \end{equation*}

**Figure 3:**
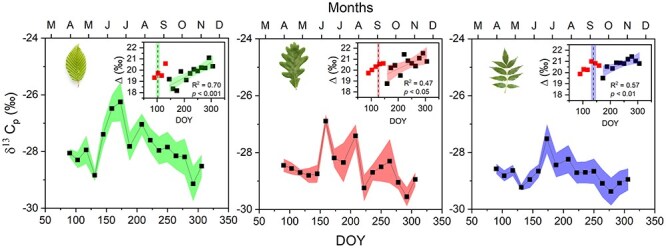
Stable carbon isotope composition of phloem contents (δ^13^C_p_) in hornbeam (green), oak (red), and ash (blue) during 2021 vegetative season. Bands indicate the standard error of the mean (*n* = 5 per species). Inset figures represent the variation of observed carbon isotope discrimination (Δ; ‰), which adjusts δ^13^C_p_ for seasonal variation in δ^13^C of the atmosphere. Bands indicate a 95% confidence interval of linear regression. Red (early season) squares on the inset graphs highlight data collected during and just after foliage expansion (see Materials and methods for details). Dashed lines within the inset graphs indicate full leafout (FF 100%), with bands representing the standard deviations.

The δ^13^C_a_ data used for Δ calculations in Eq. (3) were obtained from the polynomial fit presented in see Fig. S3 as Supplementary data at *Tree Physiology* Online. The ISO/SF method required the determination of daily values of Δ. Linear regression was used to model the data, excluding those values collected prior to full leaf FF (shown in red in Fig. 3). This approach was based on the assumption that carbohydrates present before this stage are likely remnants of the previous year’s photosynthates.

Subsequently, the ratio of the internal to ambient concentration of CO_2_ (*C_i_*/*C_a_*) was derived from (Farquhar et al. 1982)


(4) \begin{equation*} C_i\!\ / C_a = \left(\Delta - a\right)/\left(b- a\right),\end{equation*}

where *a* is the fractionation caused by the diffusion of CO_2_ in air (4.4‰); *b* is the fractionation resulting from the active site of the Rubisco enzyme (27‰). Subsequently, a proxy for iWUE (*C_a_* – *C_i_*) was derived from


(5) \begin{equation*} C_a- C_i = C_a\times \left(1- C_i\! / \!C_a\right). \end{equation*}

The net photosynthesis rate of individual trees/species (A_ISO/SF_, g C day^−1^ tree^−1^) was then calculated as:


(6) \begin{equation*} {\mathrm{A}}_{ISO/ SF}={g}_{\mathrm{tree}}\times 0.625\times \left({C}_a-{C}_i\right)\times \frac{M_C}{10^6}\ \end{equation*}

with 0.625 representing the ratio of the diffusivities of CO_2_ to H_2_O in air, and M_C_, the molar mass of C (12 g mol^−1^).

Finally, the rate of stand-scale photosynthesis (GPP_ISO/SF_, g C m^−2^ day^−1^) per species was estimated by multiplying the average per-tree photosynthetic rate of each species by the respective number of trees of each species per hectare (refer to Table S1 as Supplementary data at *Tree Physiology* Online and Fig. 2).

### Statistics

Given that the assumptions of homogeneity and normality were not met for the photosynthesis rates among the species, the Kruskal–Wallis test was employed to analyze variance and identify differences across species. When significant differences were found, a *post*-*hoc* Dunn’s test was used for pairwise comparisons. To evaluate the agreement between the methods, we employed linear regression for the analysis spanning the whole year. All statistical analyses in this study were conducted with a significance level of α = 0.05. The data analyses and visualization were performed using R statistical software (ver. 4.2; R Core Team, Vienna, Austria) and OriginPro software (OriginLab Corporation, MA, USA).

## Results

### Microclimatic conditions

In the study year, 2021, meteorological conditions were mostly normal. The average temperature was 10.4 °C and total precipitation was 480 mm (Fig. 1). These values are marginally warmer (by 0.8 °C) and drier (20 mm) than the long-term average (1961 to 2017). The only unusual weather was in April and May, when it was 2 °C colder than the long-term average (Fig. 1a).

The daytime vapour pressure deficit (VPD_D_) was consistently high from early June to mid-August (Fig. 1b). Precipitation was relatively low in the first part of the year (January to May), but SWC did not begin to fall until the end of May. The summertime fall in SWC was briefly alleviated by abundant rainfall in mid-July and again in mid-August (Fig. 1d). However, after this short relief, the SWC continued to fall until late September (Fig. 1c).

### Species-specific sap flow and leaf phenology

Variations in vegetative leaf phenophases and sap flow patterns were observed among the species within the study year (Fig. 2). In the spring, BB and FF occurred first in hornbeam, subsequently in oak, and lastly in ash. A similar order was observed in autumn, with leaf coloring (LC) occurring first in hornbeam, then oak, and finally in ash. However, compared with the other two species, ash exhibited much more rapid leaf senescence. Similarly, hornbeam initiated transpiration earliest, followed by oak and ash (Fig. 2). For hornbeam, nearly 2 weeks passed between full leafout (FF100%) and the beginning of transpiration, but oak and ash began to transpire as soon as they were fully leafed out (Fig. 2). The delay in hornbeam occurred during a period when nighttime temperatures were still falling nearly to 0 °C (Fig. 1). Hornbeam and ash achieved their maximum sap flow around mid-June (day of year (DOY) 170), whereas oak’s peak came slightly later, around early August (DOY 215; Fig. 2). Maximum flow rates were similar in ash and hornbeam and somewhat higher in the oak. After the leaf area was fully developed (FDLA), sap flow rates began to decrease across all species, with a particularly rapid decline following the first appearance of leaf coloring (LC 10%); this decline was especially noticeable in ash.

### Isotope composition and discrimination

Seasonal variation in the isotopic composition of phloem contents (δ^13^C_p_) differed among species (Fig. 3). At the beginning of the growing season, before full leafout, hornbeam presented higher δ^13^C_p_ compared with the other species. The discrimination data, which were corrected for seasonal variation in the δ^13^C of atmospheric CO_2_, showed a distinct decrease in all species 4-5 weeks after full leafout. Disregarding this early period, a distinct negative trend was observed in δ^13^C_p_ (Fig. 3) concurrent with a significant positive trend in discrimination (Δ; see inset in Fig. 3). The three species displayed similar Δ trends, with statistical analysis confirming no significant difference in the slopes among species (*F* = 0.40, *P* = 0.672). Despite similar seasonal trends, discrimination values varied among species, with hornbeam at 19.7‰ ± 0.25 SEM significantly different from the other tree species (*P* < 0.05). Meanwhile, oak and ash, at 20.5‰ ± 0.25 SEM and 20.7‰ ± 0.17 SEM, respectively, were not significantly different from each other (*P* = 0.768).

### Species differences in per-tree photosynthesis and contribution to canopy GPP

**Figure 4:**
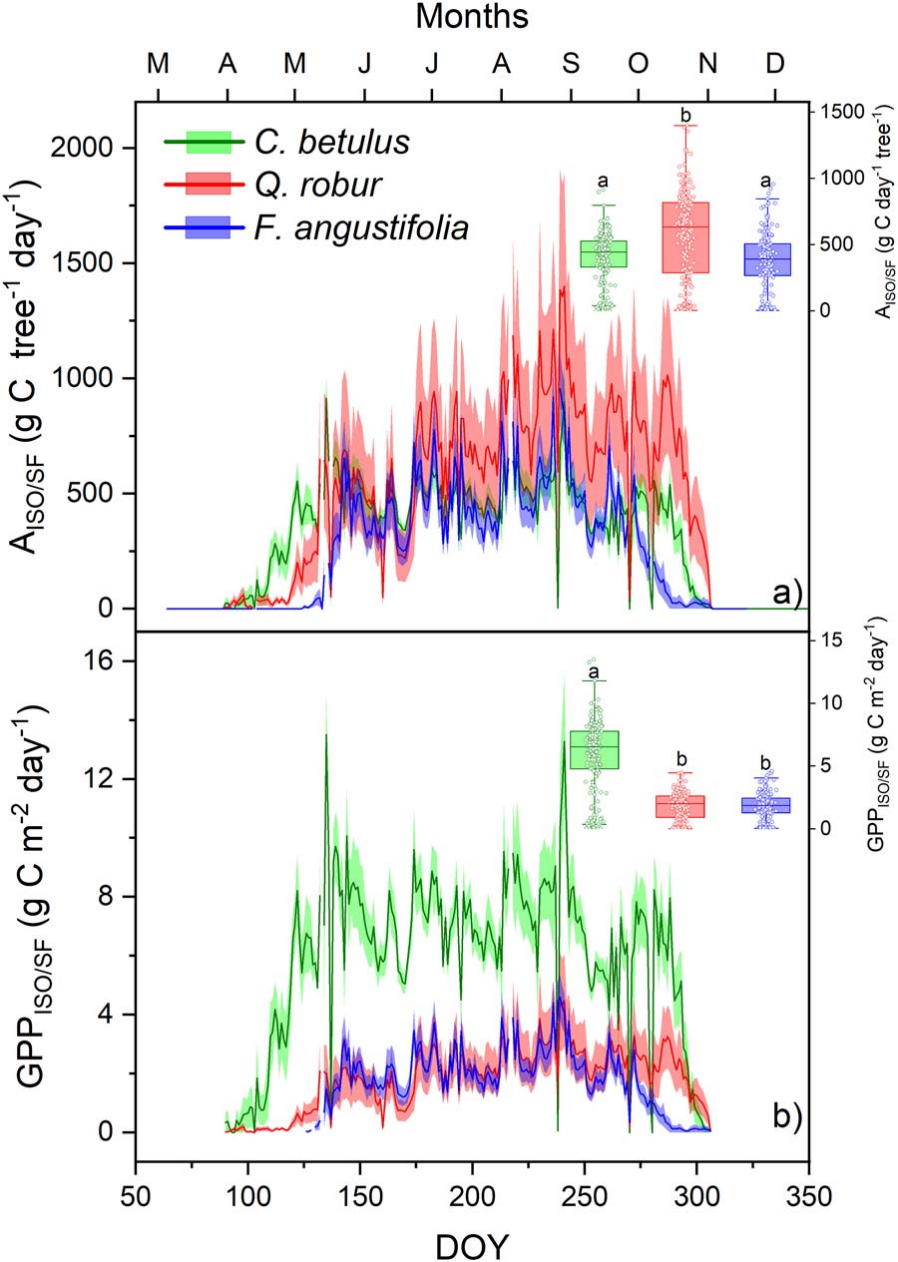
(a) Daily photosynthesis rate (A_ISO/SF_; g C tree^−1^ day^−1^) and (b) scaled up GPP (GPP_ISO/SF_; g C m^−2^ day^−1^) in hornbeam (green), oak (red) and ash (blue) during 2021 vegetative season. Color bands denote the standard error of mean (*n* = 5).Inset boxplots illustrate the distribution of mean daily A_ISO/SF_ and GPP_ISO/SF_ across the species, with letters indicating significantly distinguishable species.

**Figure 5:**
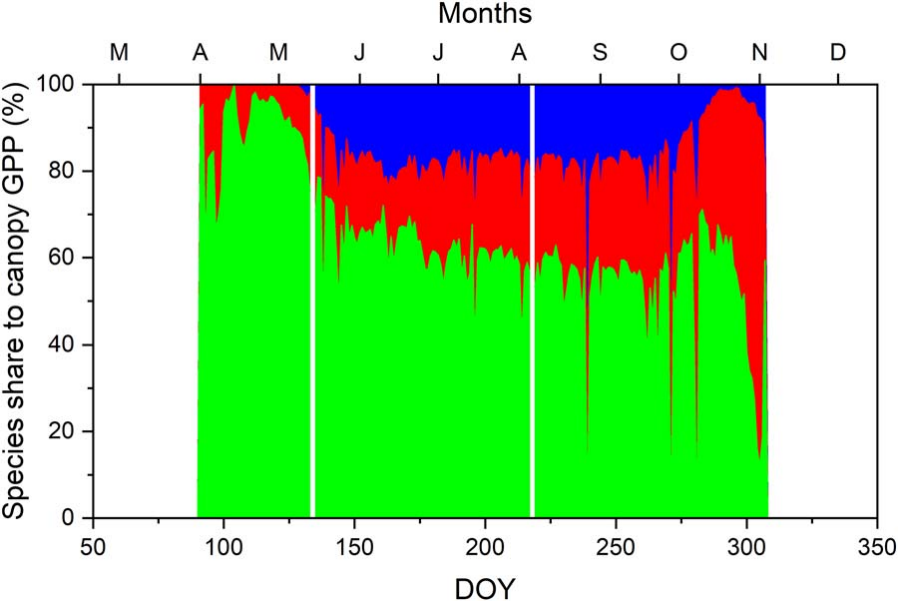
Proportional contribution of each species to daily canopy GPP, where canopy GPP was estimated as the daily sum of GPP_ISO/SF_ over the three tree species. In the graph, green (at bottom) represents hornbeam, red (middle) represents oak, and blue (top) represents ash.

Seasonal dynamics of A_ISO/SF_ (per tree) and GPP_ISO/SF_ (per species) varied among species (Fig. 4). In spring, carbon fluxes began earlier in the understory hornbeam compared with the oak and ash and dominated canopy GPP until DOY 120 or so (Fig. 5). All species began to increase their carbon fluxes in response to rising temperatures and foliage development, steadily maintaining this upward trend until the end of April. However, the cold temperatures of April and May (Fig. 1) triggered a noticeable decrease in carbon fluxes in these months, especially in May (Fig. 4). All species resumed the increase in the latter part of June, reaching a peak in August before a subsequent decline. Hornbeam and oak managed to sustain relatively high carbon fluxes until the end of the season even though the oak and ash had leafed out above the hornbeam and it showed signs of partial leaf senescence (see Fig. S1 as Supplementary data at *Tree Physiology* Online). As a consequence, hornbeam represented a high proportion of canopy GPP until the very end of the growing season (Fig. 5). In contrast, ash rapidly declined following the initiation of leaf senescence. At the level of individual trees, oak exhibited significantly higher values (*P* < 0.001) compared with hornbeam and ash, which did not significantly differ (*P* = 0.47) between them (Fig. 4a). On the other hand, at the level of species contribution to canopy GPP, hornbeam displayed significantly higher values (*P* < 0.001), while oak and ash were not significantly different (*P* = 0.24; Fig. 4b).

**Figure 6:**
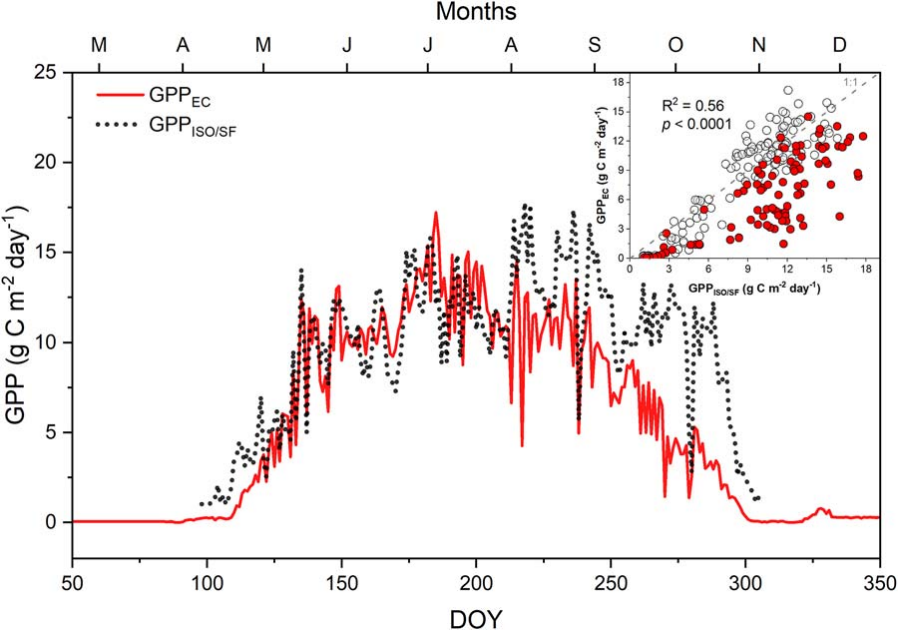
Daily estimations of GPP_EC_ (solid red) and GPP_ISO/SF_ (dashed dark gray). Inset chart compares daily estimates of GPP by EC and phloem isotope/sap flow methods. The statistics displayed within the inset graph apply to the entire observed period. The open symbols represent the period prior to DOY 212 (R^2^ = 0.85), while the red-filled symbols denote the period afterward (R^2^ = 0.60).

The GPP values estimated by the EC and ISO/SF methods were in good agreement during 2021 (Pearson’s *r* = 0.76, *P* < 0.0001), with the regression producing a significant linear fit (R^2^ = 0.56, *P* < 0.0001; Fig. 6). However, the match was better before DOY 212, after which the ISO/SF method began to yield higher, but still correlated values compared to EC. As a consequence, cumulative estimates by the ISO/SF and EC methods agreed remarkably well (*P* = 0.273) until DOY 212 with totals of 1066 g C m^−2^ year^−1^ and 992 g C m^−2^ year^−1^, respectively, after which the GPP_ISO/SF_ method overestimated relative to the GPP_EC_ method (Fig. 6). Over the whole period, GPP_ISO/SF_ was 28% higher than GPP_EC_, at 2071 g C m^−2^ year^−1^ vs 1606 g C m^−2^ year^−1^, respectively.

## Discussion

### GPP_ISO/SF_ and GPP_EC_ method comparison

Our study used a completely independent method to estimate GPP and compared it to the conventional EC method. Although the ISO/SF method has been used before, ours was the first that used the THB approach to measure sap flow. Also for the first time, the ISO/SF method was applied to a deciduous broadleaf forest ecosystem, where dramatic phenological changes might challenge the ISO/SF method. The methods yielded highly similar seasonal patterns until the end of July. The ISO/SF method yielded higher, but still correlated values starting around DOY 212 (July 31) and continuing to the end of the photosynthetic season; this late-season discrepancy yielded higher annual totals compared to GPP_EC_ estimates (Fig. 6). Given that the measurements are completely independent, yet show close agreement in the early season, this improves confidence in the robustness of the methodologies during that period. At the same time, the discrepancies late in the growing season raise questions about what may differ at that time. In the text that follows, we first review uncertainties and limitations of each method, then compare species differences and seasonal dynamics.

### Uncertainties and limitations

#### Eddy covariance

Canopy decoupling has been a particular concern with regard to the use of EC to estimate quantitatively accurate fluxes in forests. For example, one previous study conducted in a complex, multilayered forest in Switzerland, which examined both below- and above-canopy fluxes, also identified decoupling issues (Paul-Limoges et al. 2017). The study found that below- and above-canopy fluxes became decoupled under full canopy closure, thus leading to unaccounted for below-canopy fluxes when measured only above the canopy. This was particularly important because under-canopy fluxes comprised primarily a net carbon source dominated by soil respiration (Paul-Limoges et al. 2017). This pattern suggests that the EC method has limitations in accurately capturing fluxes moving from the surface to the atmosphere in the presence of a forest canopy. However, a previous study at our site found that decoupling occurred frequently but contributed little bias to estimates of net ecosystem exchange (Kowalska et al. 2022), because the floodplain is so flat that it offers no pathways for advective CO_2_ transport off-site. Consequently, although decoupling occurs, its primary effect would be to delay the appearance of some fluxes at the height of the sensor. It would have little effect on longer term carbon budgets derived via EC at this site.

#### Sap flow

Our results emphasize the significant contribution of sap flow measurements to the GPP_ISO/SF_ estimations, as highlighted in previous studies that have employed this approach (Hu et al. 2010; Klein et al. 2016). One criticism of the GPP_ISO/SF_ method is that there have been problems with the calibration of sap flow data at other sites (Klein et al. 2016; Vernay et al. 2024). These problems are so far mostly confined to the widely used Granier Heat-Dissipation (GHD) method (Steppe et al. 2010; Vernay et al. 2024). In contrast, the sap flow measurements in our study are based on the THB method (Čermák et al. 1973, 2004; Kučera et al. 1977; Sakuratani 1981). The THB method does not require calibration in the sense of adding empirical coefficients of sapwood depth or wound coefficients (Urban et al. 2012). Notably, the THB method has been used for calibration of other methods (i.e. GHD) in the field (Lundblad et al. 2001; Klein et al. 2014, 2016). Unlike the GHD sensor, it is not based on point temperature measurements that must be scaled across the sapwood, but on heating a known volume of xylem (Tatarinov et al. 2005). The value given from the measurement is not the density or velocity of the flow but the mass of sap flowing in that volume (i.e. sap flow rate; Flo et al. 2019). This direct xylem heating of the THB method renders it suitable even for large flows, which may occur in species with large-diameter vessels (i.e. ring-porous angiosperms; Kučera et al. 2020). Such species, which include the oak and ash reported here, have shown greater bias than other species when the GHD method is employed to estimate sap flow density (Yi and Xu 2023).Thus, the THB method has a strong physical and mathematical basis (Tatarinov et al. 2005), reducing one of the sources of error that arises with other methods, and was necessary for the application of the ISO/SF method to these species.

#### Stable isotopes and intrinsic water-use efficiency

Several previous studies have used δ^13^C to estimate iWUE. In these studies, δ^13^C was determined by sampling either from the foliage (Hu et al. 2010), from a complex mixture of stemwood and foliage (Klein et al. 2016; Oulehle et al. 2023) or from phloem contents (Vernay et al. 2020, 2024). We inferred iWUE from the isotopic composition of phloem contents. This approach provides a weighted integration of whole-canopy photosynthesis, where the weighting is based on net photosynthetic rates (Ubierna and Marshall 2011). Moreover, the estimate is updated as phloem contents are replaced. We observed a distinct break in the seasonal progression of isotopic discrimination in the phloem contents of all three species 4-5 weeks after the new leaves appeared, perhaps as the new photosynthate was redirected downward after supporting the burst of new growth after budbreak (inset, Fig. 3). After the break, phloem Δ increased linearly in a way that we were unable to explain with weather data (not shown). Some reports claim that the isotopic composition is modified, as phloem contents are mixed and transported down the tree (Offermann et al. 2011; Bögelein et al. 2019); however, the largest difference is between leaves and branch phloem (Bögelein et al. 2019).The discrepancy in GPP that arose between ISO/SF and EC after Day 212 was roughly consistent in direction and size with predictions from these studies, leading us to speculate that downstream modification of phloem δ^13^C was restricted to this late season period. If so, one must be careful with the ISO/SF estimates during this time. Further studies of seasonal trends in vertical phloem δ^13^C would be valuable as a test. However, even if the method is only unbiased before the end of July, it is still valuable as a test of the GPP_EC_ estimates during that period.

Some previous authors have corrected the iWUE estimates for mesophyll conductance (g_mes_). Both Klein et al. (2016) and Vernay et al. (2020) explicitly accounted for this parameter, which provides a bias adjustment for the inference of iWUE from δ^13^C. In the present study, we assumed that this adjustment is built into the 27‰ photosynthetic discrimination (Eq. (5); Cernusak et al. 2013). This assumption might miss the seasonal variation in g_mes_, observed in pine (Stangl et al. 2022), and we are eager to measure it in future work. We discuss its possible consequences below.

#### The role of understory vegetation

The GPP_ISO/SF_ method provides photosynthetic estimates only for the trees whose sap flow is measured, whereas GPP_EC_ quantifies carbon fluxes of the entire ecosystem, introducing a source of potential bias. For instance, the contribution of understory vegetation to overall ecosystem exchange can be substantial, as demonstrated in previous studies (Jarosz et al. 2008; Tian et al. 2021; Marshall et al. 2023). However, these studies reported results from relatively simple, monospecific forest ecosystems with a leaf area index of 2 to 3 m^2^ m^−2^, whereas our study site exhibited a higher leaf area index of 6 to 7.5 m^2^ m^−2^ during the growing season (Kowalska et al. 2020). Moreover, the understory contributions in the previous studies were only 6% to 7% of total GPP (Tian et al. 2021; Marshall et al. 2023). At our site, as overstory leaves emerge, PPFD in the understory decreased sharply (see Fig. S4 as Supplementary data at *Tree Physiology* Online), reducing the light available for photosynthesis. Consequently, the carbon fluxes measured by the EC tower beneath the canopy were predominantly controlled by respiration, with photosynthesis by understory vegetation remaining negligible (see Fig. S4 as Supplementary data at *Tree Physiology* Online). Similar results were obtained in multilayered forest in Switzerland, where under-canopy fluxes were dominated by soil respiration (Paul-Limoges et al. 2017). Thus, at our study site, the majority of carbon flux could be attributed to the trees. Although the GPP_ISO/SF_ method must be downward biased by the neglect of understory photosynthesis, the effect here should have been negligible.

### Species differences

Hornbeam leafed out nearly a month earlier than the other two species (Fig. 2), allowing it to maximize carbon gain before the main canopy formed (Augspurger et al. 2005). Additionally, its leaves continued to transpire well into the end of the season, even as they yellowed, while the transpiration of ash leaves gradually diminished even when they remained green (Fig. 2). In fact, the hornbeam contributes a remarkably large proportion to the GPP of the whole stand throughout photosynthetic period—even after the dominant oaks and ashes have leafed out (Fig. 5). Although its per-tree photosynthetic rates are smaller than those of oak (Fig. 4a), its high abundance more than compensates. In terms of GPP, one might well term this a hornbeam-dominated forest.

This disparity suggests an opportunity to enhance carbon sequestration through improved forest management. For instance, carefully balancing the density of hornbeam, which consumes large amounts of water while producing rather little biomass (Szatniewska et al. 2022), could more effectively allocate soil moisture resources among oaks and ashes, perhaps enhancing carbon sequestration and forest productivity (Fig. 4a).

## Conclusion

In this multispecies forest, the two methods, isotope/sap flow and EC, agreed well in their canopy photosynthesis (GPP) estimates from late spring until late July. The isotopic estimates of intrinsic water-use efficiency varied less over the growing season than the sap flux estimates, which rose from essentially zero as the leaves emerged. The THB method for measuring sap flow required no calibration to achieve this agreement across all species, improving upon the thermal dissipation estimates that have previously been used for this approach to estimating GPP. More work is required on the extent to which phloem contents reflect canopy photosynthesis, but this work should be focused on the latter part of the growing season. Hornbeam was by far the dominant contributor to canopy GPP throughout the growing season, which is somewhat surprising considering its subordinate position in the canopy.

## Supplementary Material

Suplementary_Data_tpae064

## Data Availability

Primary datasets on phloem isotopes, tree-level sap flow, and EC-derived GPP are openly available in the ASEP public repository (operated by the Czech Academy of Sciences): https://doi.org/10.57680/asep.0586665.
