# Supplementary material for: Disaggregation of canopy photosynthesis among tree species in a mixed broadleaf forest
Source: Tree Physiol. 2024 Jun 12;44(7):tpae064. doi: 10.1093/treephys/tpae064 (PMC11240116; doi:10.1093/treephys/tpae064)
Supplement: Suplementary_Data_tpae064 [file suplementary_data_tpae064.docx]

**Supplementary data**

Disaggregation of canopy photosynthesis among tree species in a mixed broadleaf forest

**Authors**

Marko Stojanović^1^, Georg Jocher^1^, Natalia Kowalska^1^, Justyna Szatniewska^1^, Ina Zavadilová^1^, Otmar Urban^1^, Josef Čáslavský^1^, Petr Horáček^1^, Manuel Acosta^1^, Marian Pavelka^1^, John D. Marshall^1,2,3,4*^

**Full addresses**

^1^Global Change Research Institute, Czech Academy of Sciences, Bělidla 4a, 603 00 Brno, Czech Republic

^2^Department of Forest Ecology and Management, Swedish University of Agricultural Sciences, Umeå, Sweden

^3^Leibniz-Zentrum für Agrarlandschaftsforschung, 15374 Müncheberg, Germany

^4^Department of Geological Sciences, Gothenburg University, Gothenburg, Sweden

***Corresponding author:**

John D. Marshall ([marshall.j@czechglobe.cz](mailto:marshall.j@czechglobe.cz))

**Table S1.** Stand structural characteristics at the end of the growing season 2020

| Tree species | No. tree ha^-1^ | DBH  (cm) | Height  (m) | BA species  (m^2^ ha^-1^) | Share in BA_stand_ (%) |
| --- | --- | --- | --- | --- | --- |
| *Carpinus betulus* | 148 | 27.7±11.2 | 23.4±6.9 | 10.41 | 34.4 |
| *Quercus robur* | 38 | 51.8±12.3 | 31.0±3.6 | 7.13 | 23.5 |
| *Fraxinus angustifolia* | 48 | 56.4 ± 9.2 | 35.9±3.4 | 12.30 | 40.6 |
| *Ulmus laevis* | 6 | 38.9±2.2 | 28.8±1.8 | 0.35 | 1.2 |
| *Acer campestre* | 4 | 19.8±7.0 | 20.0±0.6 | 0.07 | 0.2 |
| *Tilia cordata* | 2 | 18.3 | 11.8 | 0.03 | 0.1 |

Mean values ± standard deviations are shown. DHB – diameter at breast height. BA – basal area

**Fig. S1.** Scatter plot illustrating the relationship between tree height and diameter at breast height (DBH) of the main tree species in the experimental study plot at the end of the growing season 2020. Logarithmic curve has been fitted to the data points to emphasize the overall trend.**Figure S2.** Frequency distribution of diameter at breast height (DBH) on main tree species in the experimental study plot at the end of the growing season 2020. The black and white asterisks indicate sampled trees for sap flow and phloem isotope.**Figure S3.** Atmospheric δ^13^ signature (δ¹³C_a_, ‰) in 2021 from the Ochsenkopf, Germany station.


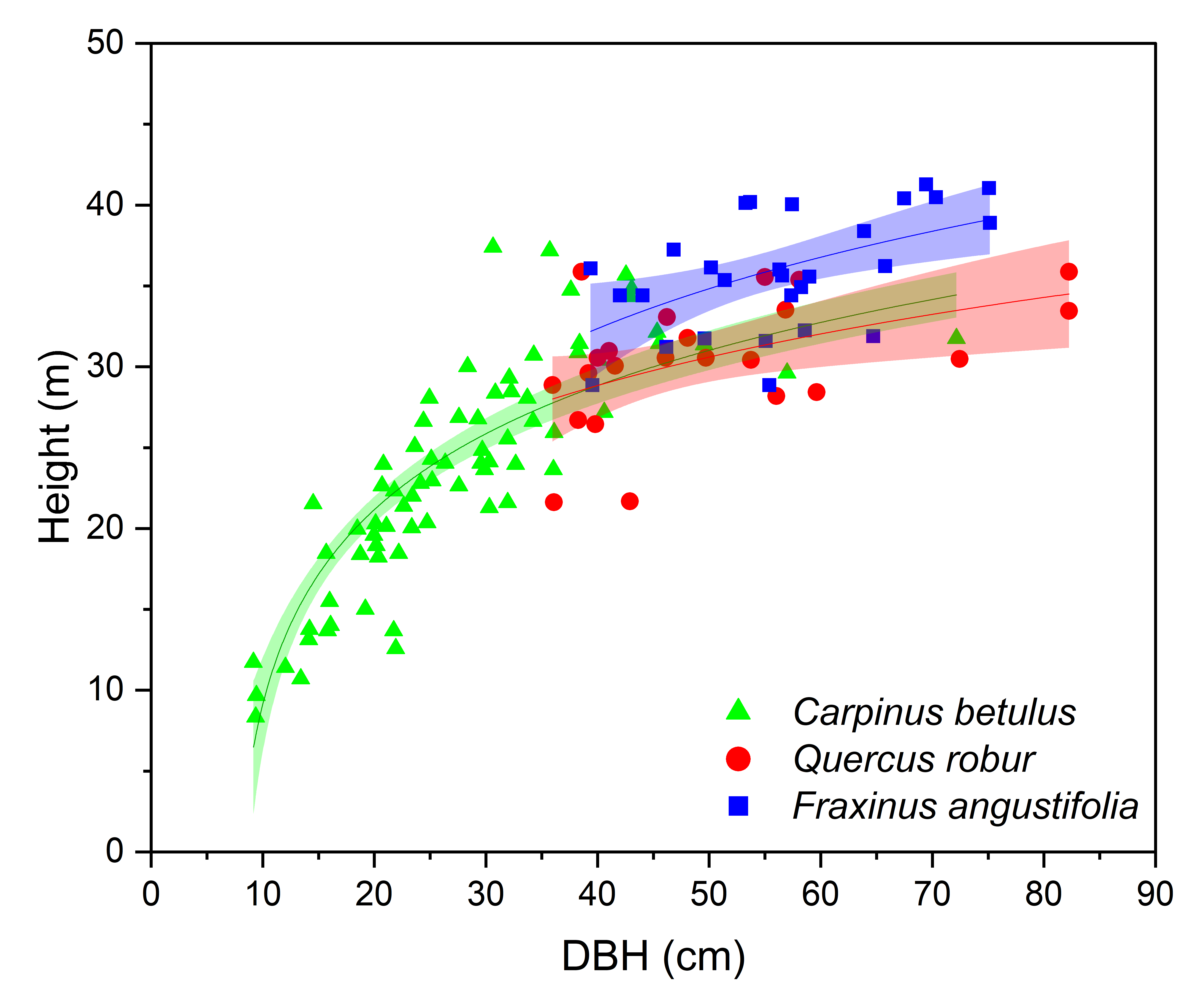

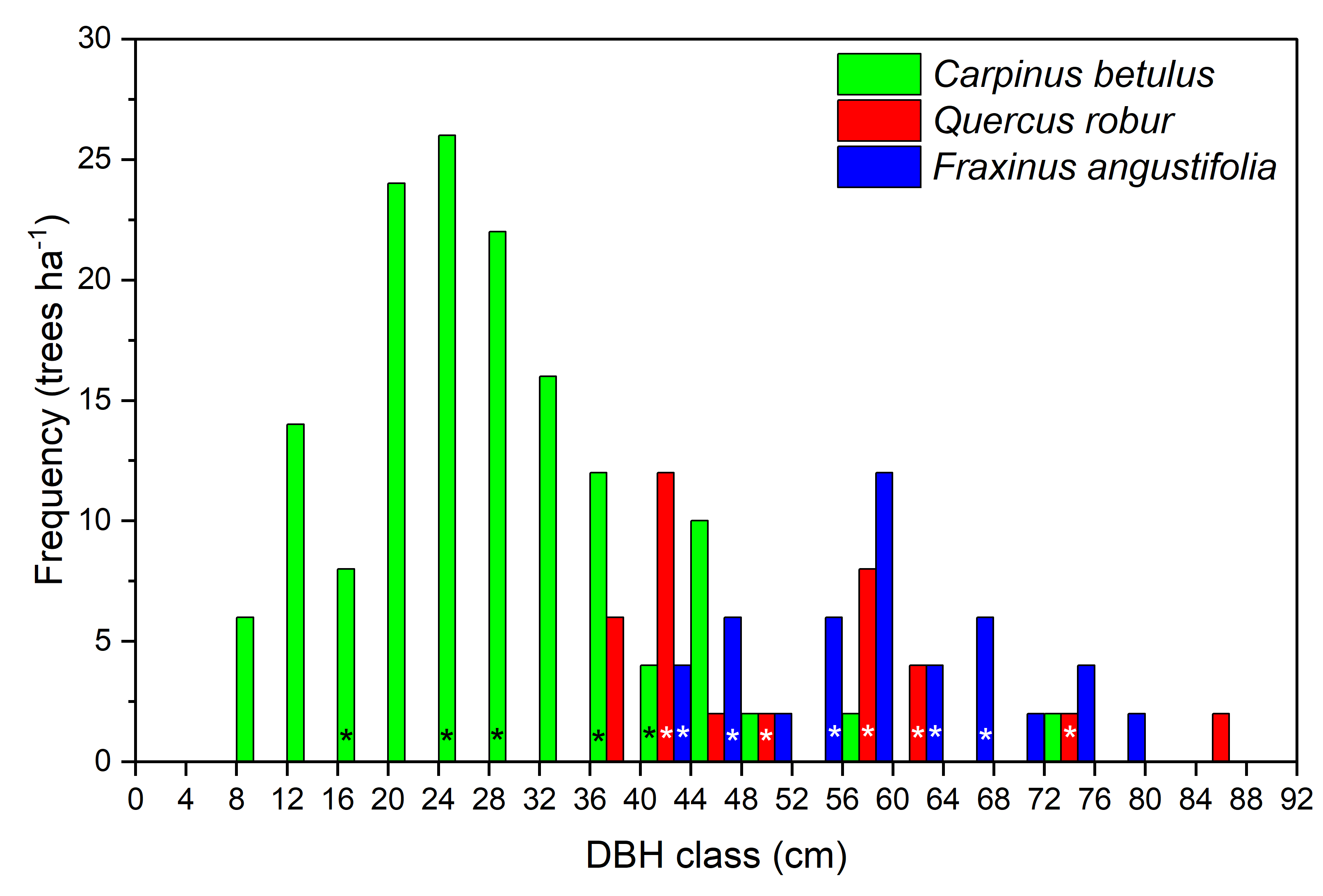

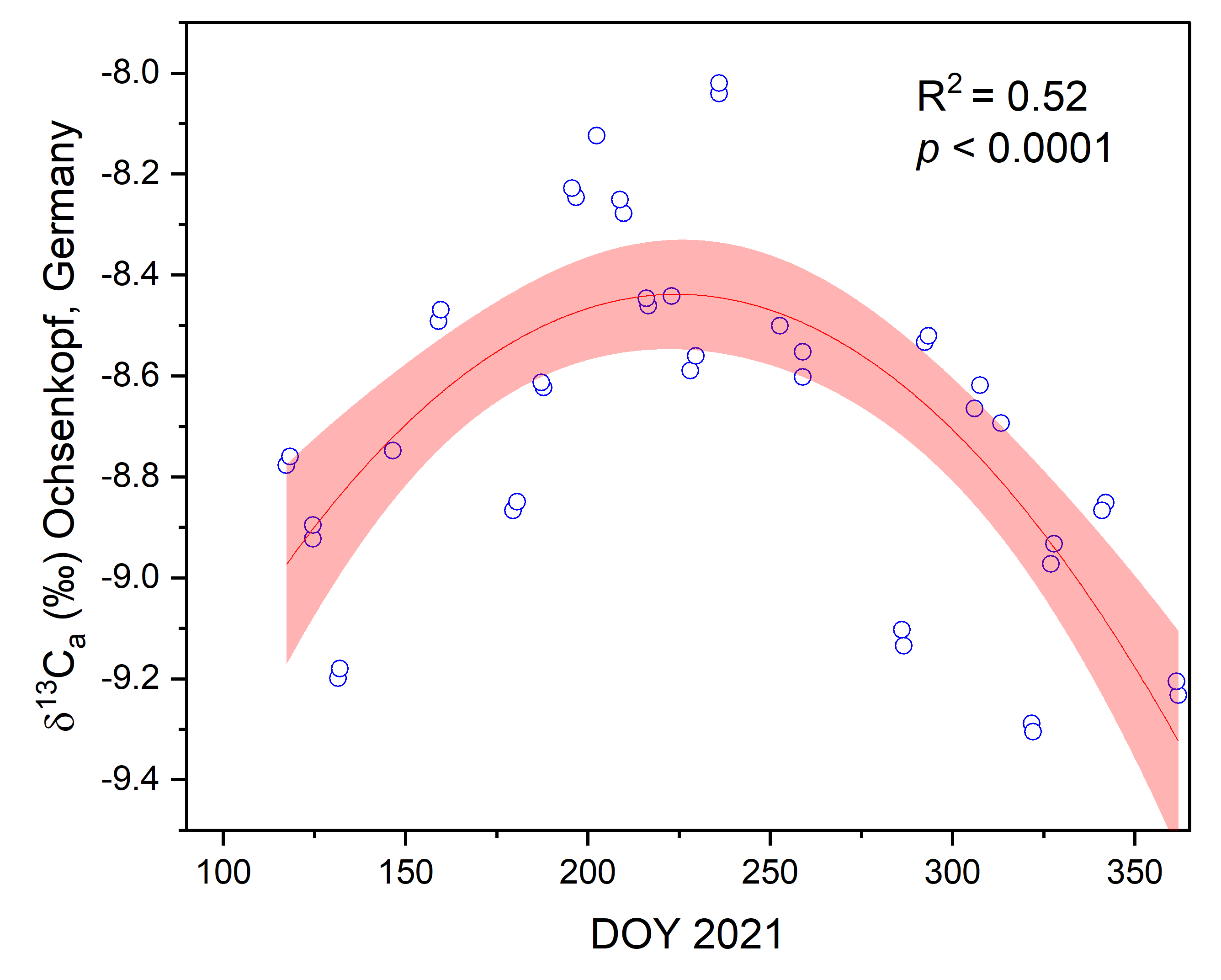


**
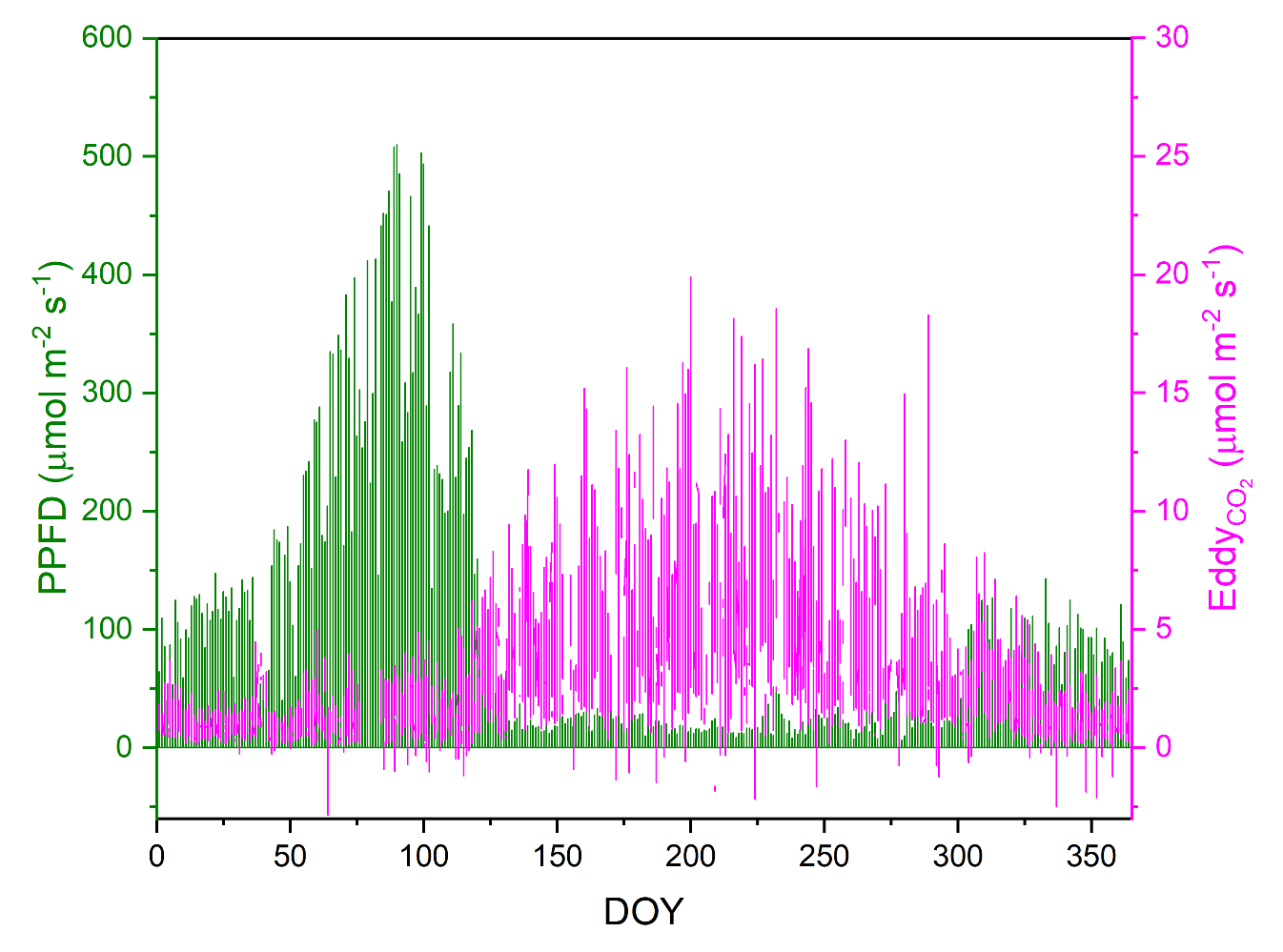
**

**Figure S4.** Photosynthetic photon flux density (PPFD; µmol m^-2^ s^-1^) and Eddy covariance CO_2_ fluxes (Eddy_CO2_; µmol m^-2^ s^-1^) measured under the canopy at 30-minute intervals.
